# Predicting Quality of Life in Parkinson’s Disease: A Machine Learning Approach Employing Common Clinical Variables

**DOI:** 10.3390/jcm13175081

**Published:** 2024-08-27

**Authors:** Daniel Magano, Tiago Taveira-Gomes, João Massano, António S. Barros

**Affiliations:** 1 Ph.D. Program in Health Data Science, Faculty of Medicine, University of Porto, 4200-319 Porto, Portugal; 2 Medical Department, BIAL-Portela & Cª., S.A., 4745-457 São Mamede do Coronado, Portugal; 3 Department of Community Medicine, Information and Decision in Health, Faculty of Medicine, University of Porto, 4200-319 Porto, Portugal; tiagogomes@med.up.pt; 4 Faculty of Health Sciences, University Fernando Pessoa, 4200-150 Porto, Portugal; 5 SIGIL Scientific Enterprises, 4076 Dubai, United Arab Emirates; 6 Department of Clinical Neurosciences and Mental Health, Faculty of Medicine University of Porto, 4200-319 Porto, Portugal; jmassano@med.up.pt; 7 Department of Neurology, Centro Hospitalar Universitário de São João, 4200-319 Porto, Portugal; 8 Department of Surgery and Physiology, Cardiovascular R&D Centre-UnIC@RISE, Faculty of Medicine, University of Porto, 4200-319 Porto, Portugal; asbarros@med.up.pt

**Keywords:** Parkinson’s disease, health-related quality of life, predictive modeling, machine learning, Parkinson’s disease questionnaire—39, PDQ-39 summary index, explainable machine learning

## Abstract

**Background**: Parkinson’s Disease significantly impacts health-related quality of life, with the Parkinson’s Disease Questionnaire-39 extensively used for its assessment. However, predicting such outcomes remains a challenge due to the subjective nature and variability in patient experiences. This study develops a machine learning model using accessible clinical data to enable predictions of life-quality outcomes in Parkinson’s Disease and utilizes explainable machine learning techniques to identify key influencing factors, offering actionable insights for clinicians. **Methods**: Data from the Parkinson’s Real-world Impact Assessment study (PRISM), involving 861 patients across six European countries, were analyzed. After excluding incomplete data, 627 complete observations were used for the analysis. An ensemble machine learning model was developed with a 90% training and 10% validation split. **Results**: The model demonstrated a Mean Absolute Error of 4.82, a Root Mean Squared Error of 8.09, and an R^2^ of 0.75 in the training set, indicating a strong model fit. In the validation set, the model achieved a Mean Absolute Error of 11.22, a Root Mean Squared Error of 13.99, and an R^2^ of 0.36, showcasing moderate variation. Key predictors such as age at diagnosis, patient’s country, dementia, and patient’s age were identified, providing insights into the model’s decision-making process. **Conclusions**: This study presents a robust model capable of predicting the impact of Parkinson’s Disease on patients’ quality of life using common clinical variables. These results demonstrate the potential of machine learning to enhance clinical decision-making and patient care, suggesting directions for future research to improve model generalizability and applicability.

## 1. Introduction

Parkinson’s Disease (PD)’s global prevalence more than doubled between 1990 and 2015 [1]. The motor symptoms associated with the second most common neurodegenerative disorder include resting tremor, rigidity, bradykinesia, and postural instability. Additionally, non-motor symptoms such as cognitive impairment, mental health disorders, sleep disturbances, pain, and sensory disturbances are also common in PD [2,3,4]. All these symptoms decrease the health-related quality of life (HRQoL) of people with PD [5].

In recent years, HRQoL has emerged as a critical outcome measure in the study of chronic diseases. HRQoL instruments, particularly those designed for specific diseases, offer valuable information on the personal effects of disease and any changes that are perceived over time. For example, in the context of PD, several disease-specific HRQoL tools have been developed recently. A comprehensive, systematic review conducted in 2002 determined that the Parkinson’s Disease Questionnaire-39 (PDQ-39) was the most suitable instrument for assessing HRQoL in the context of PD. The PDQ-39 was chosen based on several factors, including its extensive testing, robust clinimetric properties, widespread use in numerous studies, and availability in numerous languages [5]. Additionally, recent studies have demonstrated its utility in various contexts, such as assessing the impact of deep brain stimulation of the subthalamic nucleus on the quality of life of PD patients [6] and validating its use in PD patients with cognitive impairment, showcasing its versatility and applicability across different patient populations [7]. Furthermore, the Italian version of the PDQ-39 has been validated, highlighting its cross-cultural applicability [8]. Research suggests that the PDQ-39 is a reliable and valid tool for assessing HRQoL in PD, as it provides a comprehensive view of the disease’s impact, extending beyond traditional clinical outcomes, and reflects the patient’s perspective, capturing their personal and subjective experience of living with the disease [9].

PDQ-39 is a commonly utilized self-report questionnaire comprised of 39 items that assess the degree of difficulty experienced by individuals diagnosed with PD across eight dimensions of HRQoL, namely mobility, activities of daily living, emotional well-being, stigma, social support, cognition, communication, and bodily discomfort [10]. The PDQ-39 Summary Index (PDQ-39 SI), which measures the overall impact of PD on a patient’s life, is a commonly utilized patient-reported endpoint in clinical trials [10,11,12]. Despite its widespread use, accurately predicting the PDQ-39 SI and, consequently, the HRQoL remains a complex and challenging task. This complexity is partly due to the subjective nature of the questionnaire, which captures individual patient experiences and perceptions that may vary widely based on personal factors, such as disease severity, duration, treatment response, individual resilience, and varying interpretations of quality of life.

Machine and statistical learning have been used in PD to predict disease progression, motor symptoms, and treatment response. For instance, Chandrabhatla et al. explored the co-evolution of machine learning (ML) and digital technologies to enhance the monitoring of Parkinson’s Disease motor symptoms, thereby improving real-time symptom tracking and management [13]. Gao et al. employed both model-based and model-free ML techniques for the diagnostic, prediction, and classification of clinical outcomes in PD, highlighting their potential in early diagnosis and personalized treatment planning [14]. Nilashi et al. evaluated the effectiveness of ensemble ML methods for predicting PD progression, demonstrating the improved accuracy and robustness of combined models [15]. Dadu et al. focused on identifying and predicting PD subtypes and progression using ML in two cohorts, which can aid in precise disease classification and management [16]. Additionally, a recent study introduced a Perceiver architecture-based multimodal ML model to estimate PD symptom severity by analyzing raw gait signals and features from Ground Reaction Force sensors [17]. However, its application in predicting HRQoL, as measured by the PDQ-39 Summary Index (SI), remains underexplored. This study sought to develop an ML model capable of predicting PDQ-39 SI in patients with PD using readily available clinical variables, such as the patient’s current PD medication, co-existing medical conditions, education level, age, and age at diagnosis. The objective of this research is to develop an ML model equipped with explainable ML techniques to enhance clinicians’ ability to predict HRQoL in PD patients. This tool is designed to provide deeper insights into the factors influencing HRQoL, thereby informing treatment decisions and ultimately improving patient care. Additionally, the explainable ML component of this study offers critical information about the variables that have a higher impact on quality of life, which can be valuable for clinicians in understanding disease progression and management.

## 2. Materials and Methods

### 2.1. Database and Data Preparation

**Figure 1:**
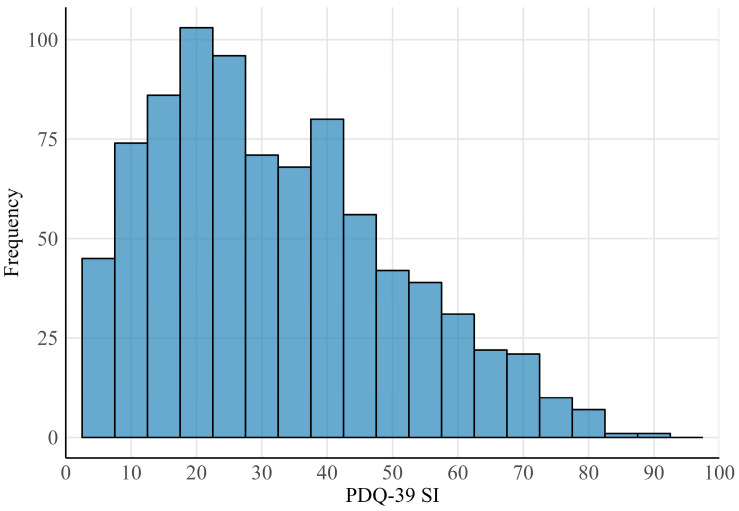
Distribution of PDQ-39 SI across 859 patients from the PRISM study.

The data for this study were sourced from the Parkinson’s Real-world Impact Assessment (PRISM) study, a cross-sectional observational research initiative. A total of 861 patients with PD and 256 caregivers from six European countries participated in the PRISM study, completing an online survey using structured questionnaires including the PDQ-39, Non-Motor Symptoms Questionnaire, and Zarit Burden Interview [18]. This survey captured a broad range of information, including medication use, comorbidities, and healthcare resource utilization [18]. After excluding two observations due to missing PDQ-39 SI scores, 859 valid entries remained for analysis. The demographic profile of the participant pool was nearly evenly split by gender: 48.5% female and 50.3% male, with a mean age of 65.0 years (Standard Deviation (SD) = 10.2 years). The average PDQ-39 SI score was 32.0 (SD = 18.2), with its distribution depicted in Figure 1. Supplementary Table S1 summarizes key variables from the PRISM dataset.

### 2.2. Data Analysis

All analyses were performed using the R statistical software (version 4.3.0) [19]. As part of our analytical approach, we utilized Factor Analysis of Mixed Data (FAMD) to explore the underlying relationships between variables within the PRISM dataset, including the PDQ-39 SI. FAMD are particularly relevant for datasets that include both quantitative and qualitative variables, as they extend the principles of principal component analysis to mixed data types. This approach allows us to reduce the complexity of the data by identifying the principal dimensions that capture the most variance. Consequently, FAMD provide a comprehensive understanding of how different variables interact and contribute to the observed patterns in our dataset. The results of the FAMD are visually depicted in Supplementary Figure S1, offering a clear illustration of the interrelations among the dataset where variables that are highly correlated cluster together, including how closely each variable correlates with the outcome variable, the PDQ-39 SI.

**Figure 2:**
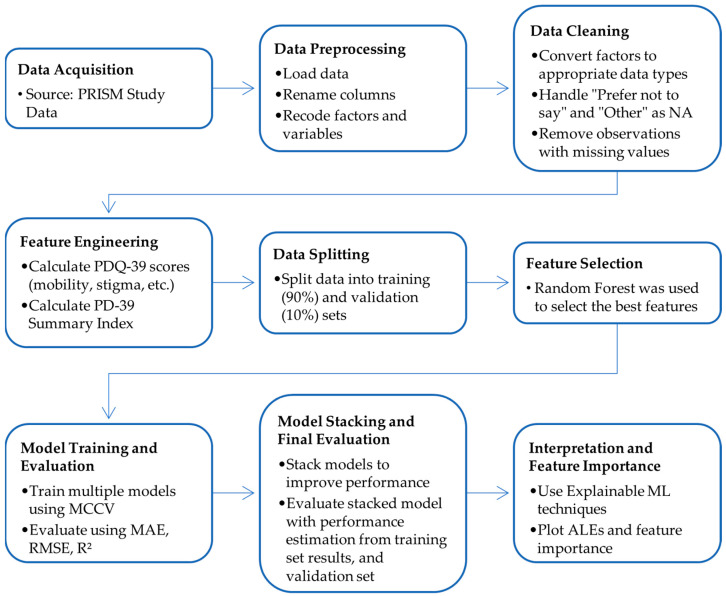
Diagram of the study process from data acquisition to data preparation, feature selection, model training, and performance evaluation.

After the FAMD analysis, the PRISM dataset was subjected to a data-cleaning process. Categorical variables with responses such as ‘prefer not to say’ or ‘other’ were treated as missing values to minimize the noise in the final model. Upon examination of the dataset (Supplementary Table S1), it was observed that it contains minimal missing values. Most of these missing values appear to be missing completely at random, with notable exceptions in the variables age at diagnosis and current medication. These exceptions suggest a missing not at random pattern, likely due to patients not recalling the age of PD diagnosis or being unaware of the therapeutic class of their current medication. Although we initially attempted to address missing values using Multivariate Imputation by Chained Equations (MICE), this approach resulted in a model with worse predictive capabilities [20]. Therefore, we only kept complete observations, resulting in a total of 627 valid entries. Subsequently, the dataset was partitioned into training (90%) and validation (10%) sets to maximize the learning capacity of our model amidst the constraints of limited sample size and high variability inherent in questionnaire-based data. This allocation allows for comprehensive model training on a diverse range of responses, critical for capturing the nuanced patterns of HRQoL assessments in PD, while still reserving a portion of the data for essential validation to assess model performance and mitigate overfitting. Figure 2 illustrates this study’s workflow, detailing each step from data acquisition to the data split and comparison of regression metrics in the training and validation sets.

### 2.3. Model Training

The models were trained on a personal computer equipped with an AMD Ryzen 7 7800X3D CPU, 32 GB of RAM, and an AMD Radeon RX 7900 XT GPU (Advanced Micro Devices, Inc., Santa Clara, CA, USA). We evaluated several feature selection methods—specifically, random forest, least absolute shrinkage and selection operator (LASSO), group LASSO, and the option of omitting feature selection—to identify the most suitable technique for our dataset. Feature selection through random forest was selected for its standout performance in enhancing the predictive accuracy of our suite of ML models. We then applied this feature selection approach in training a diverse range of ML models, namely linear regression, boosted generalized linear model, extreme gradient boosting with linear models and decision trees, ridge regression, neural network regression, LASSO regression, random forest, k-nearest neighbors, support vector machines with radial basis function and polynomial kernel, and Gaussian processes (linear, polynomial, and radial kernels), incorporating essential pre-processing steps such as centering and scaling with the caret package [21]. To accurately train and evaluate the performance of these models within the context of a dataset characterized by limited size and high variability, Monte Carlo Cross-Validation (MCCV) was employed with 500 repetitions for each model. This approach was chosen for its effectiveness in mitigating data variability, thereby ensuring a more reliable and stable assessment of the models’ generalizability.

### 2.4. Model Stacking

To further improve the performance of our predictive model, we implemented a stacking approach using an ensemble of the previously trained ML models. Stacking is an advanced ensemble method that combines multiple predictive models to enhance overall accuracy. It utilizes a meta-model that optimally combines the predictions from several base models trained on the same data. This technique leverages the strengths and compensates for the weaknesses of individual models, thereby increasing the robustness and reliability of predictions [22].

We started by assessing the correlation among these models to create an ensemble characterized by both strong individual performance and minimal intermodel correlation. We then trained several ensembles using MCCV and experimented with different numbers of repetitions (100, 250, 500, and 1000) to assess both stability and performance. For the stacking mechanism, we tested both random forest and extreme gradient boosting with decision trees (XGBTree) as techniques. These were selected for their demonstrated capability to effectively handle complex data patterns. This stacked ensemble approach allowed us to enhance the model’s ability to generalize across different patient profiles and improve the accuracy of predicting the PDQ-39 SI.

### 2.5. Model Evaluation

The performance of the models was evaluated using multiple metrics. The Mean Absolute Error (MAE) measures the average magnitude of the errors in predictions, providing a straightforward measure of the average error magnitude per prediction. The Root Mean Squared Error (RMSE) offers insight into the accuracy of the model by quantifying the square root of the average squared differences between the predicted values and actual observations. Lastly, the Coefficient of Determination (R^2^) reflects the proportion of variance in the dependent variable that is predictable from the independent variables, thereby indicating the explanatory power of the model. These metrics collectively provide a comprehensive assessment of model performance.

The ensemble model that emerged as the most effective incorporated the pre-trained ML models: Gaussian process with a Radial Basis Function kernel and random forest. This ensemble model was trained using MCCV with 1000 repetitions, with XGBTree serving as the stacking method. MCCV was utilized both to train and to estimate the performance of the ensemble model on the training dataset, while the validation set was reserved for the final assessment to evaluate overfitting susceptibility and ensure an unbiased evaluation of the model’s generalizability.

### 2.6. Interpretable Machine Learning Analysis

To unveil the importance and effect of each feature on the predictive model, we employed interpretable ML techniques, particularly focusing on Permutation Feature Importance (PFI). Utilizing the iml package in R, this analysis facilitates a deeper understanding of the behavior of our final ensemble ML model [23]. The core concept of PFI is to measure the increase in the model’s prediction error after the permutation of each feature’s values, which effectively breaks the relationship between the feature and the true outcome. This process provides a clear indication of a feature’s importance; a significant increase in error indicates that the model relied heavily on the feature for prediction, whereas no change in error suggests that the feature was not utilized by the model for prediction [24].

The process of feature importance quantification involves permuting the values of each feature and assessing the change in the model’s performance using MAE as the chosen metric, due to its lower sensitivity to outliers and variability within the dataset. This procedure was carried out for each feature individually and repeated 100 times to obtain a more robust estimate, with the results averaged thereafter.

## 3. Results

### 3.1. Performance Metrics

**Figure 3:**
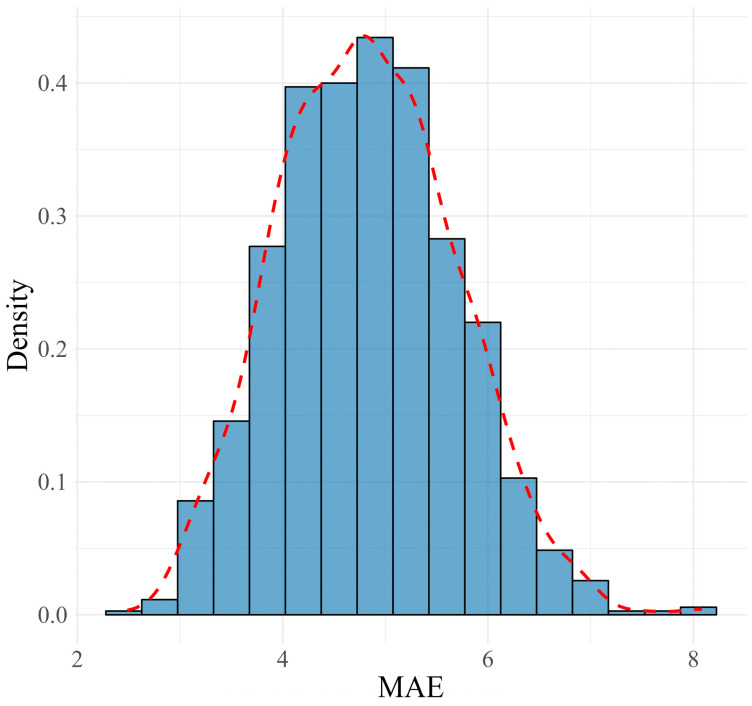
Distribution and density curve of MAE values from 1000 MCCV repetitions, demonstrating the consistency and reliability of the predictive model in training.

**Figure 4:**
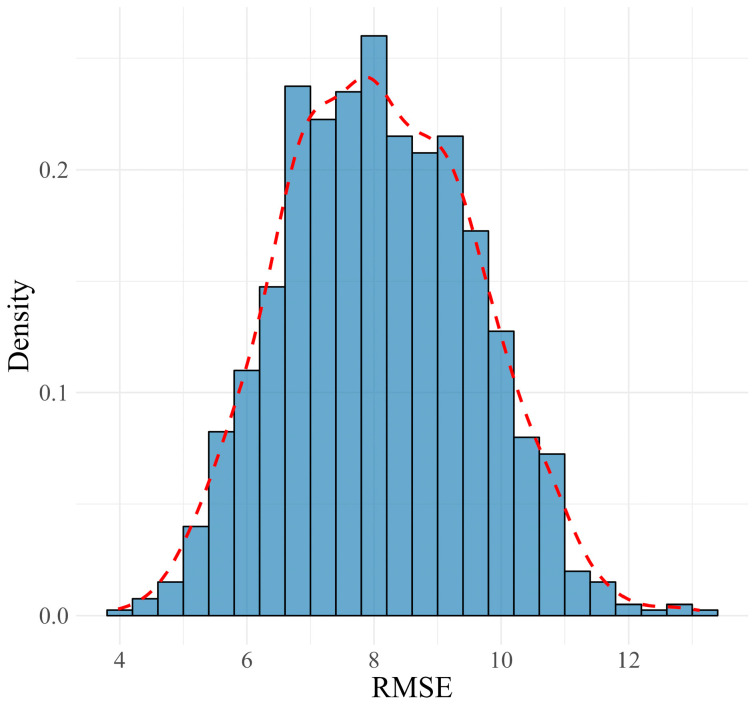
Distribution and density curve of RMSE values from 1000 MCCV repetitions, assessing the model’s accuracy and stability during the training phase.

**Figure 5:**
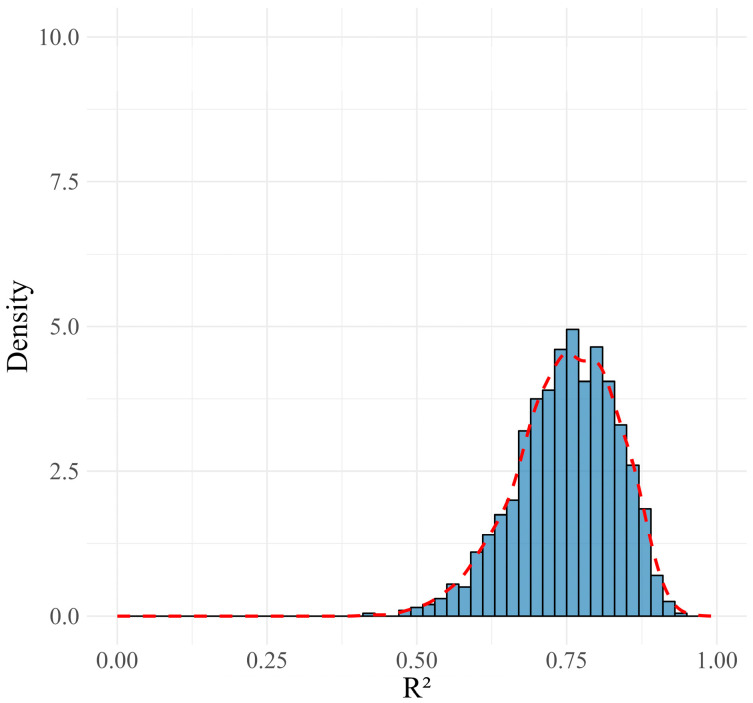
Distribution and density curve of R^2^ values from 1000 MCCV repetitions, showcasing the predictive power and fit of the model to the training data.

In the training set, the final ensemble model achieved an MAE of 4.82, an RMSE of 8.09, and an R^2^ value of 0.75, indicating a strong fit to the data. The distributions of MAE, RMSE, and R^2^ metrics from the 1000 MCCV repetitions approximated normal distributions, as shown in Figure 3, Figure 4 and Figure 5. These distributions suggest that the model’s performance is consistent and reliable across different subsamples of the training data, reflecting its stability. When applied to the validation set, the model exhibited moderate variations in performance metrics, with an MAE of 11.22, an RMSE of 13.99, and an R^2^ value of 0.36, indicating the model’s reasonable predictive capability on unseen data. A summary of these results is presented in (Table 1).

**Figure 6:**
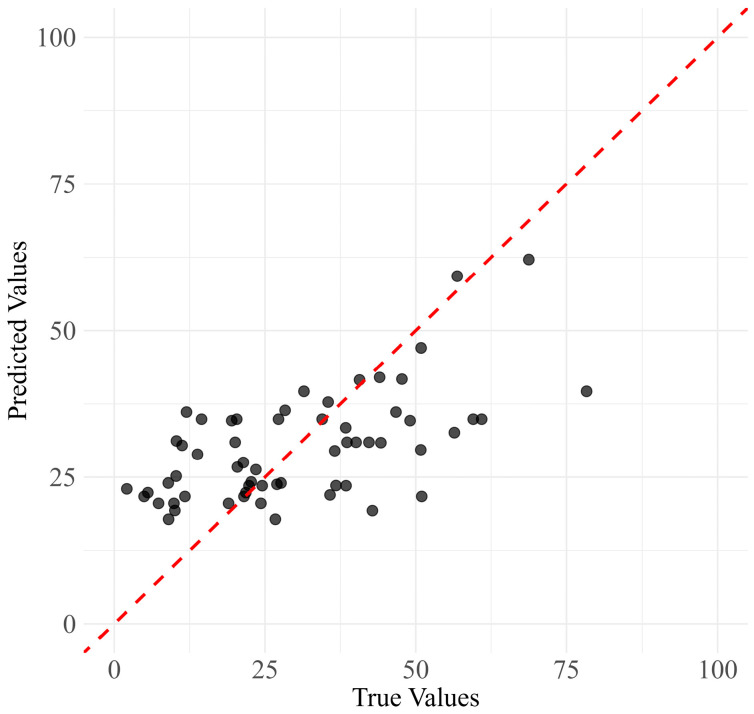
Scatter plot showing the relationship between actual PDQ-39 SI scores (true values) and model predictions (predicted values). The black dots represent individual predictions, and the red dotted line represents the line of perfect prediction where predicted values would equal true values.

**Figure 7:**
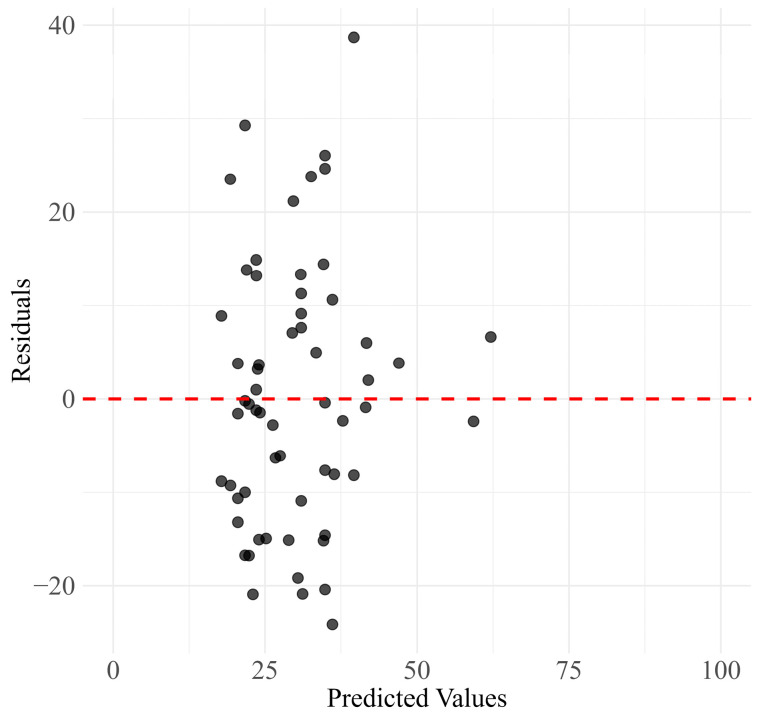
Scatter plot of residuals (errors) against predicted values, indicating prediction accuracy and error distribution. The black dots represent the residuals (actual value − predicted value), and the red dashed line represents the zero-error line.

The True vs. Predicted Values plot (Figure 6) demonstrates the relationship between the actual PDQ-39 SI scores and the scores predicted by the model. The plot suggests that the model tends to slightly overpredict at lower values and underpredict at higher values, indicating areas for potential refinement. The Residuals vs. Predicted Values plot (Figure 7) shows that while most residuals are clustered around zero, there is a slight spread, suggesting occasional larger prediction errors.

### 3.2. Interpretation

The ensemble model demonstrated strong predictive capabilities on the training dataset, as indicated by the high R^2^ and low MAE values. However, the model performance on the validation set showed moderate variation. Although the model remains a promising tool for estimating PDQ-39 SI scores, these variations suggest areas for future research and model refinement. The observed variations in the validation set may be attributed to the limited size of the dataset and the inherent variability of HRQoL measures, both of which warrant further investigation. The diagnostic plots suggest that while the model generally performs well, addressing the slight overprediction at lower values and underprediction at higher values could enhance its accuracy, aligning with the variability observed in the validation set. The variables selected through feature selection for their significant impact on PDQ-39 SI predictions are listed in Table 2. Feature importance and effects.

**Figure 8:**
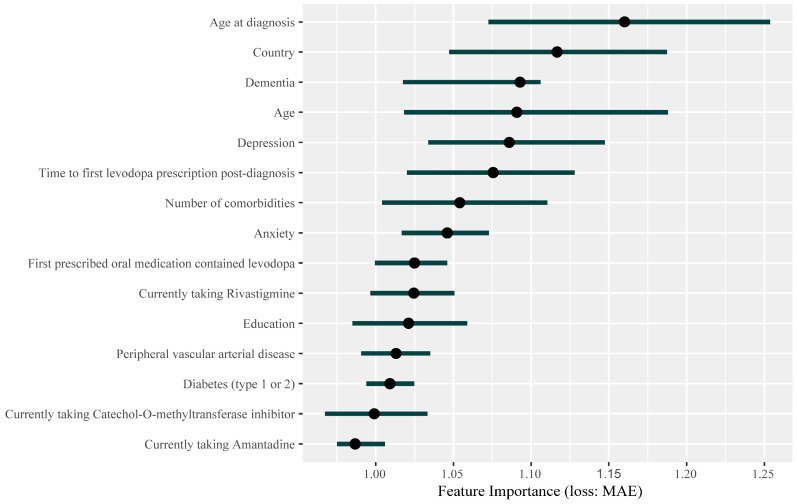
Demonstration of the feature importance of clinical variables in predicting PDQ-39 SI.

The interpretable ML analysis using the iml package in R yielded substantial insights into the significance of the individual features within our predictive model. Table 2 encapsulates the importance of each feature along with the associated 90% confidence intervals, as derived from Permutation Feature Importance, which quantifies the decrease in model accuracy when each feature’s data is randomly shuffled. Predominantly, age at diagnosis, patient’s country, dementia, and patient’s age emerged as the most important features affecting PDQ-39 SI predictions. A graphical representation of feature importance was generated to provide a holistic view of each feature’s contribution to the model’s predictive performance (Figure 8).

**Figure 9:**
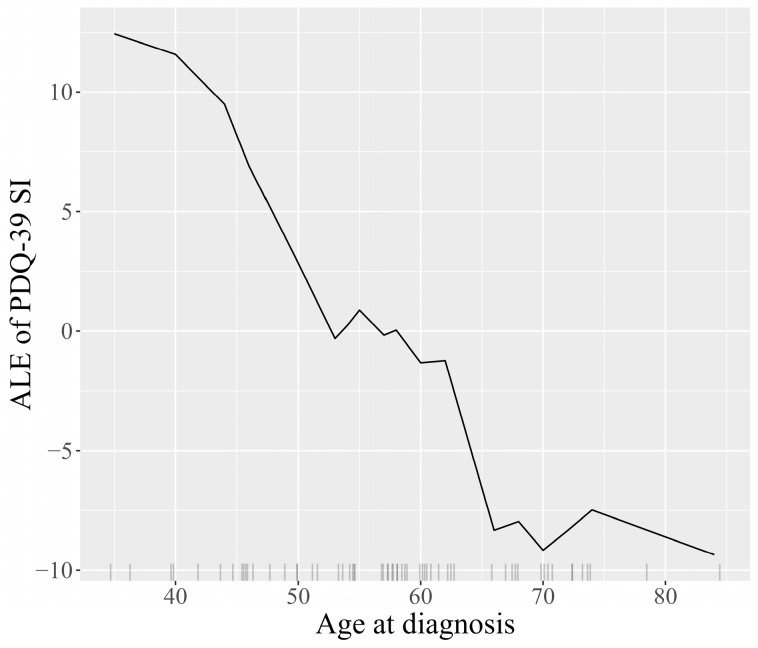
ALE plot illustrating the effect of age at diagnosis on PDQ-39 SI predictions, showing how earlier or later disease appearance impacts the quality of life in PD patients.

**Figure 10:**
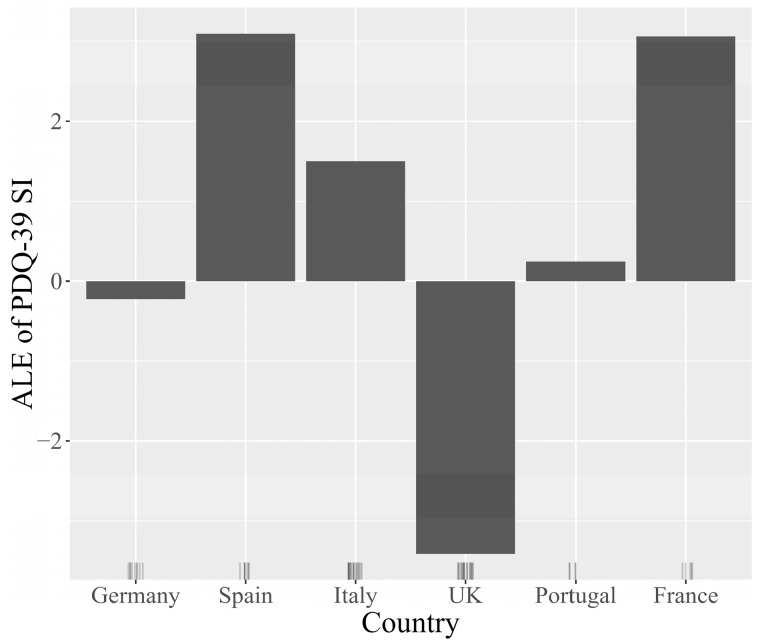
ALE plot illustrating the effect of the patient’s country of residence on PDQ-39 SI predictions, highlighting the variations in the quality of life among PD patients across different countries.

**Figure 11:**
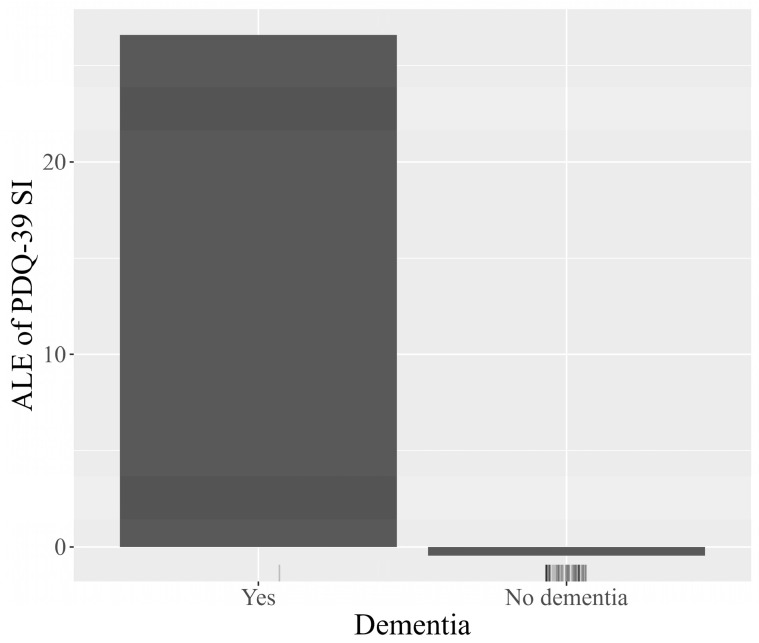
ALE plot demonstrating how the presence of dementia affects PDQ-39 SI scores predicted by the model, emphasizing its considerable negative impact on the quality of life in PD patients.

**Figure 12:**
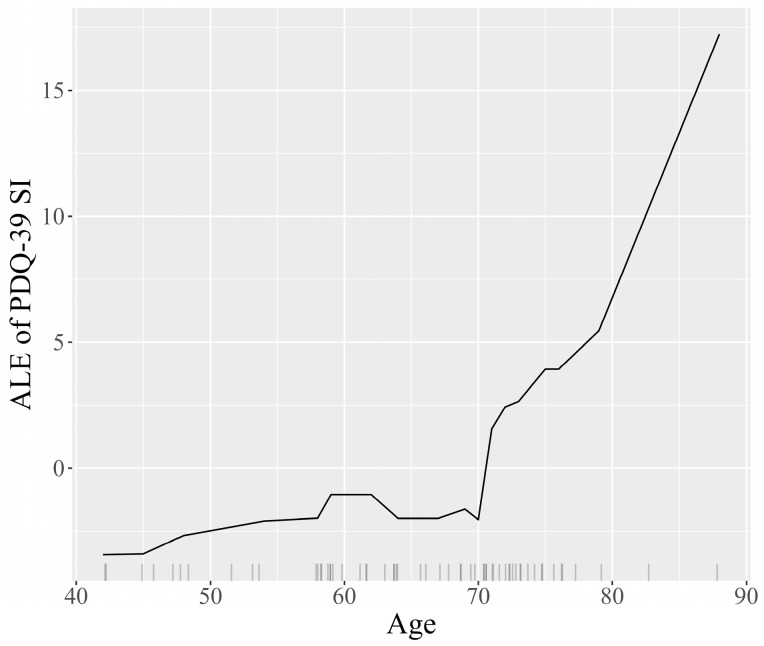
ALE plot detailing the impact of age on PDQ-39 SI predictions, illustrating the direct influence of age increments on the PDQ-39 SI scores predicted by the model.

Transitioning to a more detailed analysis, the Accumulated Local Effects (ALE) plots facilitate visual exploration of the behavior of the model, underscoring how the prediction shifts locally with variations in each feature. The plots for the features age at diagnosis, dementia, patient’s country, and patient’s age are represented in Figure 9, Figure 10, Figure 11 and Figure 12. The marks beneath the *x*-axis represent the distribution of each feature where observations were obtained. In the case of categorical variables such as dementia and the patient’s country, the marks were automatically spaced to simplify the visual interpretation. For the features age at diagnosis and patient’s age, regions with sparse or no points suggest caution in interpretation due to insufficient data.

The ALE plot for age at diagnosis (Figure 9) indicates that the earlier the disease appears, the higher the scores predicted by the model on the PDQ-39 SI, and therefore the more likely the patients are to have a lower quality of life, which is consistent with disease progression. The ALE plot for the patient’s country (Figure 10) indicates a moderate impact on the predictions of PDQ-39 SI, with the United Kingdom showing a slightly more negative impact on the outcome. The ALE plot for dementia (Figure 11) demonstrates the significant negative impact of this comorbidity on the PDQ-39 SI predictions, highlighting its burden on PD patients. The ALE plot for the patient’s age (Figure 12) demonstrates a consistent trend, indicating a direct influence of age on PDQ-39 SI predictions. This interpretive analysis not only enhances the comprehensibility of our predictive model but also extends the foundation for a more informed evaluation of the features affecting the PDQ-39 SI.

## 4. Discussion

### 4.1. Principal Results

The primary achievement of this study is the successful development and validation of an ensemble ML model tailored to predict the PDQ-39 SI score in patients with PD. The model’s strong performance in the training set, particularly its high R^2^ value, underscores its ability to account for a significant proportion of the variance in PDQ-39 SI scores. This is a noteworthy advancement given the complex interplay of factors that can influence the quality of life in patients with PD. However, it is important to note that the significant difference in R^2^ values between the training and validation sets indicates a potential issue of overfitting. The R^2^ value in the training set was substantially higher than that in the validation set, suggesting that the model may have learned patterns specific to the training data that do not generalize well to unseen data. This is common in ML applications and serves as a valuable indicator of areas that may benefit from further research and optimization. Future work should focus on addressing this overfitting issue by exploring techniques such as regularization, cross-validation, and the inclusion of more diverse training data. Additionally, model simplification and the use of ensemble methods with a more rigorous selection of base models may help enhance generalizability.

The model’s performance in the validation set, although not as strong as that in the training set, still suggests that it holds promise as a tool for estimating PDQ-39 SI scores in a clinical setting. Despite the inherent uncertainty in predictions of patient-reported outcome measures, the model provides valuable insights into disease progression and highlights the potential impact of clinical variables on the quality of life in PD patients. This indicates that the model could become a useful decision-support tool, with further refinement and validation needed to enhance its reliability and applicability.

The feature importance analysis conducted in this study provides a granular understanding of the variables that significantly impact the PDQ-39 SI score. This approach not only predicts the PDQ-39 SI score but also elucidates the underlying factors contributing to the model’s predictions, offering a more comprehensive insight into the determinants of HRQoL in patients with PD. The notable importance of variables such as age at diagnosis, dementia, and patient’s age in our model underlines the well-documented interplay among these factors and PD, as they are highly correlated with disease progression. Age is a recognized risk factor for PD, and both the prevalence and severity of the disease notably increase with age, underscoring the progressive nature of PD. The significant association between age and PD is evident from various epidemiological studies that show an exponential increase in PD prevalence from the age of 60 years onwards, reaching a peak in the age group of 70–79 years [25]. Additionally, an early appearance of the disease (represented by the feature ‘age at diagnosis’) is associated with a greater impact on the quality of life, highlighting the significant burden of the disease when it manifests early. Furthermore, dementia is a common comorbidity in patients with PD, often exacerbating the severity of the disease and the patient’s quality of life. The prevalence of dementia in PD patients ranges between 24% and 31%, with the risk increasing substantially with age [26].

Our model’s emphasis on age at diagnosis, dementia, and patient’s age as significant predictors of the PDQ-39 SI reflects the clinically observed interactions between these variables and PD symptom severity. This alignment with established clinical knowledge not only enhances the validity of our model but also underscores the potential utility of our model in clinical settings. Utilizing these critical predictors, healthcare providers can anticipate the likely trajectory of PD patients’ quality of life, aiding in the development of personalized treatment plans. It is also worth noting that other variables, such as anxiety, education, and some comorbidities, were identified as relevant by our feature selection method but did not show as high importance in the final model’s predictions. These variables might still be important for adjusting the model to control for confounding effects and improve overall accuracy, even though they do not individually have strong predictive power [27].

Although the model does provide important information on the features affecting the PDQ-39 SI, it is also important to highlight that the predictive capabilities still demonstrate some variation, especially in the validation set. This could indicate that while the clinical variables are useful, the features available in the dataset do not entirely capture the PDQ-39 SI. The observed variability suggests that there are other influential factors impacting the PDQ-39 SI that are not represented in the current dataset. As such, future research should explore the inclusion of additional variables and data sources that may better reflect the complexities of HRQoL in PD patients. This would potentially enhance the model’s accuracy and provide a more comprehensive tool for clinical application.

### 4.2. Limitations

One of the key limitations of our study was the small sample size. This could have contributed to the observed variations in performance metrics between the training and validation sets as small sample sizes can significantly impact ML performance estimates [28]. Additionally, we decided to exclude incomplete data and only use complete observations, resulting in a total of 627 valid entries. This decision was necessary because data imputation negatively impacted model performance. Future research should aim to address these limitations by incorporating larger and more diverse datasets, which could enhance the model’s generalizability and robustness. Exploring different imputation methods or techniques to handle missing data more effectively might also improve model performance.

### 4.3. Comparison with Prior Work

Our study diverges from the approach taken by Candel-Parra et al. for predicting the PDQ-39 SI score in patients with PD. Specifically, Candel-Parra et al. utilized classification models to categorize the PDQ-39 SI score into binary categories (above or below 40), whereas our study employed regression models to predict the actual score value [29]. This distinction is particularly significant given the absence of established cut-off values for the PDQ-39 SI score. Classification models, such as those used by Candel-Parra et al., require such cut-offs to categorize outcomes, introducing potential arbitrariness and statistical power loss when no clinically validated thresholds exist [29].

In contrast, our regression model predicts the actual PDQ-39 SI score, providing a more nuanced and precise assessment of a patient’s quality of life. Treating the PDQ-39 SI scores as a continuous variable aligns our model more closely with the actual behavior of the scores, enhancing its applicability and interpretability in clinical settings. Furthermore, our use of explainable ML techniques allows clinicians to simulate disease progression and understand which variables are influencing the quality of life, providing actionable insights that can inform treatment decisions and patient management. This feature is particularly valuable, as it enables healthcare providers to tailor interventions specifically to the factors that have the greatest impact on a patient’s HRQoL, such as early diagnosis, dementia, and patient’s age.

## 5. Conclusions

The ensemble model developed in this study is a promising tool for healthcare providers to predict the HRQoL of patients with Parkinson’s Disease, thereby aiding in personalized treatment strategies. Given that PDQ-39 SI is commonly used as an endpoint in clinical trials, the ability to predict this score could offer valuable insights for trial design and patient management. By leveraging regression models, our approach offers a nuanced understanding of a patient’s quality of life, filling a gap in existing research methodologies.

While the PDQ-39 is a reliable and valid tool for assessing HRQoL in PD patients, it primarily serves as a cross-sectional assessment. In contrast, our predictive model enhances the utility of the PDQ-39 by providing dynamic predictions of the PDQ-39 SI score based on readily available clinical variables. This predictive capability allows for the simulation of disease progression and the identification of key factors influencing HRQoL over time, such as age at diagnosis, dementia, and patient’s age. Consequently, clinicians can gain deeper insights into how individual patient characteristics and changes in clinical status impact the quality of life, enabling more personalized and proactive management strategies. The integration of explainable ML techniques further distinguishes our model by offering transparency into the prediction process, thereby empowering healthcare providers to understand and trust the model’s recommendations. The practical feature of utilizing clinical variables that are typically available at the moment of use, as emphasized in a recent critique of predictive models, enhances the usability and real-world applicability of our model, setting it apart from models that depend on variables that are not readily accessible in the clinical context [30].

Although the model’s performance on the validation set exhibited moderate variation, it sets the stage for future research in aspects such as feature engineering, hyperparameter optimization, and incorporation of additional clinically relevant variables. Moreover, the model’s performance is likely to improve with a larger sample size, given the inherent variability of patient-reported outcomes, such as those measured by the PDQ-39 SI. Future work should also address the potential overfitting suggested by the difference in R^2^ values between the training and validation sets.

While the model does provide important insights into the features affecting the PDQ-39 SI, it is essential to recognize that the predictive capabilities still demonstrate some variation, especially in the validation set. This variability suggests that the current dataset’s features do not entirely capture the PDQ-39 SI, indicating that additional variables and data sources might be necessary to better reflect the complexities of HRQoL in PD patients. Future research should explore these additional factors to enhance the model’s accuracy and provide a more comprehensive tool for clinical application.

The potential impact of the model on clinical practice and clinical trials underscores its value as a significant contribution to the field. Furthermore, the broader implications of this work have the potential to significantly influence how healthcare providers approach PD treatment, thereby contributing to more personalized and effective care.

## Figures and Tables

**Table 1:** Results.

Metric	Training Set	Validation Set
MAE	4.82	11.22
RMSE	8.09	13.99
R^2^	0.75	0.36

**Table 2:** Selected Features and their importance in PDQ-39 SI predictions ^a–c^.

Feature	Importance (0.05)	Importance	Importance (0.95)	Permutation Error
Age at diagnosis	1.07	1.16	1.25	13.02
Country	1.05	1.12	1.19	12.53
Dementia	1.02	1.09	1.11	12.26
Age	1.02	1.09	1.19	12.24
Depression	1.03	1.09	1.15	12.19
Time to first levodopa prescription post-diagnosis	1.02	1.08	1.13	12.07
Number of comorbidities	1	1.05	1.11	11.83
Anxiety	1.02	1.05	1.07	11.74
First prescribed oral medication contained levodopa	1	1.02	1.05	11.5
Currently taking Rivastigmine	1	1.02	1.05	11.5
Education	0.98	1.02	1.06	11.46
Peripheral vascular arterial disease	0.99	1.01	1.04	11.37
Diabetes (type 1 or 2)	0.99	1.01	1.02	11.32
Currently taking Catechol-O-methyltransferase inhibitor	0.97	1	1.03	11.21
Currently taking Amantadine	0.98	0.99	1.01	11.07

^a^ The importance of a feature is quantified by permuting the values of the feature and measuring the change in the model’s performance against a baseline using MAE. The displayed results include the estimated importance values alongside their 90% confidence intervals, represented by the lower (Importance (0.05)) and upper (Importance (0.95)) limits. ^b^ A larger permutation error indicates higher feature importance. ^c^ The process was executed for each feature and repeated 100 times to obtain a more robust estimate, with the results averaged.

## Data Availability

The data used in this study were obtained from the Parkinson’s Real-world Impact Assessment (PRISM) database (www.prism.bial.com), accessed on 18 February 2022.

## Supplementary Materials

The following supporting information can be downloaded at: https: //www.mdpi.com/article/10.3390/jcm13175081/s1, Table S1: Descriptive Statistics of the PRISM Database. Figure S1: Factor Analysis of Mixed Data.
