# Supplementary material for: Predicting Quality of Life in Parkinson’s Disease: A Machine Learning Approach Employing Common Clinical Variables
Source: J Clin Med. 2024 Aug 27;13(17):5081. doi: 10.3390/jcm13175081 (PMC11396193; doi:10.3390/jcm13175081)
Supplement: Supplementary file 1 [file jcm-13-05081-s001.zip › Supplementary Table S1 - Descriptive Statistics of the PRISM Database.pdf]

Supplementary Table S1. Descriptive Statistics of the PRISM Database

Characteristics	Overall (N=859)
Demographics	
Age, y	
Mean (SD)	65.0 (10.2)
Median [IQR]	65.0 [58.0;72.0]
Min, Max	[31.0;91.0]
Missing	6 (0.7%)
Gender	
Female	417 (48.5%)
Male	432 (50.3%)
Missing	10 (1.2%)
Country	
France	63 (7.3%)
Germany	92 (10.7%)
Italy	264 (30.7%)
Portugal	79 (9.2%)
Spain	149 (17.3%)
UK	212 (24.7%)
Education	
University	342 (39.8%)
No University	498 (58.0%)
Missing	19 (2.2%)
Comorbidities	
Anxiety	136(15.8%)
Asthma	49(5.7%)
Cancer	43(5.0%)
COPD	21(2.4%)
Dementia	36(4.2%)
Depression	187(21.8%)
Diabetes (type 1 or 2)	55(6.4%)
Gastric ulcer	47(5.5%)
Heart issues	73(8.5%)
High blood pressure	217(25.3%)
Kidney disease	16(1.9%)
Liver disease	12(1.4%)
Peripheral vascular arterial disease	31(3.6%)
Rheumatic diseases	90(10.5%)
Stroke	17(2.0%)

Characteristics	Overall (N=859)
PD Medication	
Amantadine	
Yes	75 (8.7%)
No	693 (80.7%)
Missing	91 (10.6%)
Anticholinergics	
Yes	16 (1.9%)
No	752 (87.5%)
Missing	91 (10.6%)
Catechol-O-methyltransferase inhibitor	
Yes	114 (13.3%)
No	660 (76.8%)
Missing	85 (9.9%)
Dopamine agonists	
Yes	396 (46.1%)
No	365 (42.5%)
Missing	98 (11.4%)
Levodopa	
Yes	656 (76.4%)
No	115 (13.4%)
Missing	88 (10.2%)
Monoamine oxidase-B inhibitor	
Yes	317 (36.9%)
No	447 (52.0%)
Missing	95 (11.1%)
Rivastigmine	
Yes	33 (3.8%)
No	739 (86.0%)
Missing	87 (10.1%)
PDQ-39 SI	
Mean (SD)	32.0 (18.2)
Median [IQR]	29.1 [17.9;43.8]
Min, Max	[1.3;89.3]

IQR, interquartile range; PD, Parkinson’s disease; PDQ-39 SI, Parkinson's Disease Questionnaire - 39 Summary Index; SD, standard deviation
