# Supplementary material for: Predicting Quality of Life in Parkinson’s Disease: A Machine Learning Approach Employing Common Clinical Variables
Source: J Clin Med. 2024 Aug 27;13(17):5081. doi: 10.3390/jcm13175081 (PMC11396193; doi:10.3390/jcm13175081)
Supplement: Supplementary file 1 [file Supplementary Figure S1 - Factor Analysis of Mixed Data.pdf › Supplementary Figure S1 - Factor Analysis of Mixed Data.pdf]

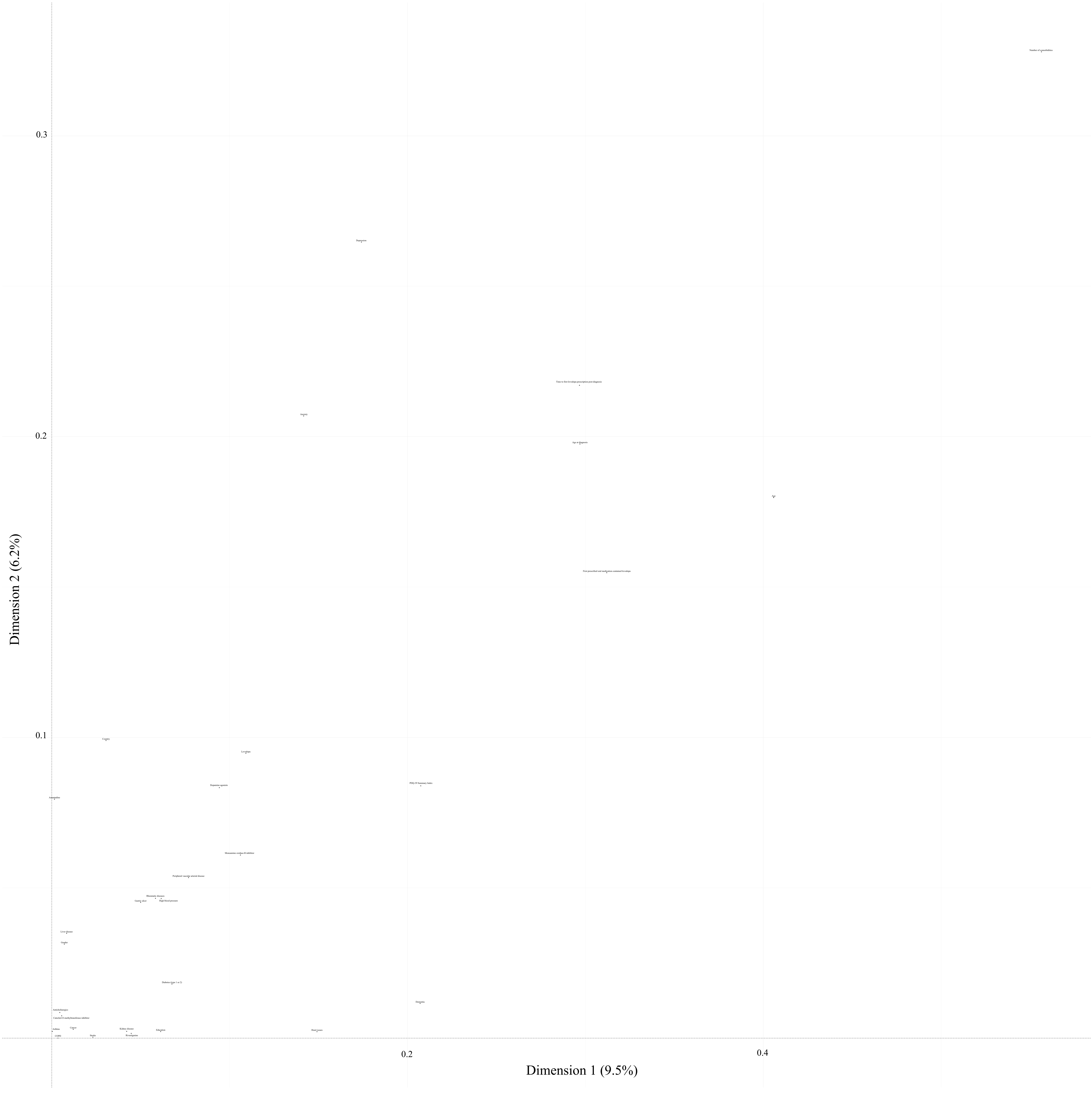
Number of comorbidities
0.3
Depression
Time to first levodopa prescription post-diagnosis
Anxiety
0.2
Age at diagnosis
Age
First prescribed oral medication contained levodopa
Dimension 2 (6.2%)
0.1
Country
Levodopa
Dopamine agonists
PDQ-39 Summary Index
Amantadine
Monoamine oxidase-B inhibitor
Peripheral vascular arterial disease
Rheumatic diseases
Gastric ulcer
High blood pressure
Liver disease
Gender
Diabetes (type 1 or 2)
Dementia
Anticholinergics
Catechol-O-methyltransferase inhibitor
Asthma
Cancer
Kidney disease
Education
Heart issues
COPD
Stroke
Rivastigmine
0.2
0.4
Dimension 1 (9.5%)
